# Microbial Composition of SCOBY Starter Cultures Used by Commercial Kombucha Brewers in North America

**DOI:** 10.3390/microorganisms9051060

**Published:** 2021-05-14

**Authors:** Keisha Harrison, Chris Curtin

**Affiliations:** 1 Department of Food Science and Technology, Oregon State University, Corvallis, OR 97330, USA; keisha.harrison@oregonstate.edu; 2 Center for Genome Research and Biocomputing, Oregon State University, Corvallis, OR 97330, USA

**Keywords:** Kombucha fermentation, acetic acid bacteria, Brettanomyces, microbiome, mycobiome, Illumina sequencing

## Abstract

Kombucha fermentation is initiated by transferring a solid-phase cellulosic pellicle into sweetened tea and allowing the microbes that it contains to initiate the fermentation. This pellicle, commonly referred to as a symbiotic culture of bacteria and yeast (SCOBY), floats to the surface of the fermenting tea and represents an interphase environment, where embedded microbes gain access to oxygen as well as nutrients in the tea. To date, various yeast and bacteria have been reported to exist within the SCOBY, with little consensus as to which species are essential and which are incidental to Kombucha production. In this study, we used high-throughput sequencing approaches to evaluate spatial homogeneity within a single commercial SCOBY and taxonomic diversity across a large number (*n* = 103) of SCOBY used by Kombucha brewers, predominantly in North America. Our results show that the most prevalent and abundant SCOBY taxa were the yeast genus *Brettanomyces* and the bacterial genus *Komagataeibacter*, through careful sampling of upper and lower SCOBY layers. This sampling procedure is critical to avoid over-representation of lactic acid bacteria. K-means clustering was used on metabarcoding data of all 103 SCOBY, delineating four SCOBY archetypes based upon differences in their microbial community structures. Fungal genera *Zygosaccharomyces, Lachancea* and *Starmerella* were identified as the major compensatory taxa for SCOBY with lower relative abundance of *Brettanomyces.* Interestingly, while Lactobacillacae was the major compensatory taxa where *Komagataeibacter* abundance was lower, phylogenic heat-tree analysis infers a possible antagonistic relationship between *Starmerella* and the acetic acid bacterium. Our results provide the basis for further investigation of how SCOBY archetype affects Kombucha fermentation, and fundamental studies of microbial community assembly in an interphase environment.

## 1. Introduction

Kombucha, an acidic beverage containing low (to null) concentrations of alcohol, is made by fermentation of sweetened tea with mixed consortia of bacteria and yeast known as a symbiotic culture of bacteria and yeast (SCOBY) [1,2]. Traditionally, SCOBY take the form of a solid-phase cellulosic pellicle, serially transferred from a finished batch of Kombucha to a new batch of tea. Formation of this solid phase is reliant upon the presence of at least one cellulose-producing acetic acid bacterium (AAB) of the *Komagataeibacter* (formerly *Gluconacetobacter*) genus [3].

Unsurprisingly then, *Komagataeibacter* is consistently cited as the most prevalent bacterial genus associated with Kombucha production and has been reported in both liquid and solid phases. Species of *Komagataeibacter* observed include *K. xylinus* [4,5], *K. rhaeticus* [6,7,8], *K. saccharivorans* [4,5], *K. intermedius* [4,8,9], and *K. kombuchae* (also known as *K. hansenii*) [4]. As well as generating the solid-phase pellicle, *Komagataeibacter* is responsible for production of organic acids integral to Kombucha’s characteristic sweet and sour flavor profile [1,10,11,12,13]. This function is presumably also performed by other genera of AAB described in SCOBY, such as *Acetobacter* [14,15], *Tanticharoenia* [16] and *Gluconobacter* [16,17], though the relative importance of these has not been elucidated. The role and prevalence of lactic acid bacteria (LAB) in Kombucha fermentation is less clear, but culture- [14] and sequencing-based [16] studies have reported genera such as *Lactobacillus*, *Lactococcus* and *Oenococcus* amongst SCOBY microbiota. The liquid phase of Kombucha fermentation, typically dominated by the same genera, possesses a more varied bacterial community. Additional minor taxa reported include *Enterobacter* [4,16], *Bifidiobacterium* [4], *Kluyvera* [16] and *Cellulosimic* [4].

By contrast, dominant yeast genera appears to be substantially more variable from study to study. *Zygosaccharomyces* [1,18], *Candida* [14], *Torulaspora* [1], *Pichia* [19,20], *Brettanomyces* [4,14,16,21], *Schizosaccharomyces* [1,19,21], *Hanseniaspora* [4], and *Saccharomyces* [1,2,18,22] are some of the genera described as important for Kombucha production. Yeast diversity in liquid and solid phases have observed the same taxa in both, but different patterns of succession during the course of fermentation [4,17]. The role of yeasts in Kombucha fermentations is to convert sugars to ethanol which is subsequently utilized by AAB [22]. Indeed, during in vitro experiments with two known Kombucha organisms, *Starmerella bacillaris* and *Acetobacter syzgii*, acetic acid was only detected in co-culture, whereas gluconic acid was detected in *A. syzgii* monoculture [23]. Variability in dominant yeasts described by past studies may reflect functional equivalence of these genera. It is also possible that earlier studies reliant upon classical microbiological methods to enumerate, isolate and identify yeast and bacteria under-sampled diversity of SCOBY communities [15,18].

DNA sequencing-based approaches to the study of microbial ecology facilitate simultaneous identification of different taxonomic groups, including those that cannot be cultured [24], shedding new light on microbial community assembly and function [25]. Increasingly applied to better understand fermented food microbial communities [26,27], to date only a handful of studies have applied these techniques to Kombucha fermentation [4,14,16]. While each has contributed a more detailed view of Kombucha SCOBY composition than prior works, they have not facilitated establishment of a consensus view of Kombucha ecology. There exists more apparent variation in SCOBY composition between these studies than within-study comparisons of SCOBY from different origins and experimental treatments, suggesting that a more comprehensive sampling of SCOBY starter cultures is required.

In this study, we utilized metabarcoding to characterize the spatial homogeneity of microbial communities in a single Kombucha production SCOBY, and then applied metabarcoding and shotgun metagenomics to broadly survey the microbial composition of SCOBY starter cultures used across the North American Kombucha industry. The objectives of this work were to evaluate whether the microbial communities assembled within Kombucha SCOBY varied spatially or across different geographic regions, and to determine the range of SCOBY archetypes used by Kombucha brewers.

## 2. Materials and Methods

### 2.1. Chemicals, Reagents and Microbiological Media

Unless stated otherwise, all chemicals and reagents were obtained from Sigma-Aldrich (St. Louis, MO, USA).

### 2.2. Microbial Media and Cultures

*Brettanomyces bruxellensis* (OSCL-Y066) was retrieved from cryogenic storage and grown on Yeast Peptone Dextrose (YPD; 10 g/L yeast extract, 20 g/L peptone, 20 g/L glucose) agar (15 g/L agar, Bioplus, Altamonte Springs, FL, USA) incubated at 30 °C for 72 h, and then single colonies were picked and transferred into 5 mL YPD broth in 15 mL ventilated centrifuge tubes (Techno Plastic Products, Trasadingen, Switzerland) incubated at 30 °C on an orbital shaking platform at 150 rpm until stationary the phase was achieved.

*Gluconobacter oxydans* (OSCL-B027) was retrieved from cryogenic storage and grown on M13 solid media (25 g/L mannitol, 5 g/L yeast extract, 3 g/L peptone, 15 g/L agar) incubated at 30 °C for 48 h, and then single colonies were picked and transferred into 5 mL M0013 broth in 15 mL ventilated centrifuge tubes (Techno Plastic Products, Trasadingen, Switzerland) incubated at 30 °C on an orbital shaking platform at 150 rpm until the stationary phase was achieved.

Cultures used for construction of qPCR standard curves were enumerated by serial dilution in sterile 0.1% peptone water and spread plating onto their respective solid media.

### 2.3. Kombucha SCOBY Starter Culture Sampling and Processing

#### 2.3.1. Sectioning of Commercial Kombucha SCOBY (Spatial Analysis Study)

A solid-phase SCOBY (approximately 86 cm in diameter) was sourced from a commercial Kombucha producer in Portland, OR. The SCOBY had previously been used to perform a 7 day Kombucha fermentation and was then stored in “starter fluid” (fermented acidic tea) at room temperature according to standard production operations. The SCOBY was transferred in a sanitized bucket to Oregon State University, and then aseptically separated into two layers (top and bottom) using a scalpel. Each layer was dissected into concentric circles (approximately 5.0 cm in width): inner, mid, and outer. Each circular section was further dissected into four (inner and mid) or eight (outer) samples. A visual representation of sampling is included in supplemental materials (Figure S1).

#### 2.3.2. Collection of Representative Commercial Kombucha SCOBY (Taxonomic Diversity Study)

Solid-phase sections of Kombucha SCOBY were obtained from commercial Kombucha brewers from October 2017 to May 2018, with the assistance of Kombucha Brewers International (KBI). Study participants were provided with a sterile sampling kit and instructions to sample from a SCOBY that had been recently used to complete a Kombucha fermentation. Timing of sampling reflected each brewer’s experience and their style-specific characteristics, such as sweet/acid balance and flavor. Participants sampled approximately 2.5 cm from an inner and an outer radial position, by cutting through all vertical layers. The duplicate samples were shipped cold to Oregon State University and stored at 4 °C prior to processing for DNA extraction. In total, 103 SCOBY samples from 29 US states and territories and 9 countries were analyzed in this study (Table S1).

#### 2.3.3. Sample Homogenization and DNA Extraction

Approximately 1 g subsections of each sample were homogenized using a VWR 200 Homogenizer (VWR, Radner, PA, USA) following a biofilm homogenization protocol [28] with modifications. Briefly, 15 mL sterile falcon tubes containing a 1:1 ratio (*w*/*v*) of SCOBY:sterile 0.1% peptone water were kept on ice and homogenized for 30 s. Prior to processing each sample, the homogenizer tip was treated for 30 s each with 2× ethanol washes (95% *v*/*v* and 70% *v*/*v*), 2× sterile water washes, and 1× DNA AWAY™ (Thermo Scientific, Waltham, MA, USA) wash. DNA from 1 mL of each homogenized sample was extracted using DNeasy Power Foods Microbial kit (QIAGEN, Hilden, Germany) according to the manufacturer’s instructions with minor modifications. In place of vortexing, the Omni Bead Ruptor 24 (Omni International, Inc., Kennesaw, GA, USA) was used with 15 s pulses at 8.00 m/s with a 55 s pause, and 10 cycles. For the Taxonomic Diversity Study, homogenates of the inner and outer sections of the SCOBY were combined prior to extraction.

All DNA extracts were quantified using the SpectraMax Quant Accuclear Nano dsDNA assay kit (Molecular Devices, San Jose, CA, USA) on the SpectraMax M2 (Molecular Devices, San Jose, CA, USA) with excitation and emission at 468 and 507 nm, respectively. ZymoBIOMICS Microbial Community Standard (Zymo Research, Irvine, CA, USA) was included as a positive control for DNA extraction. DNA extracts were stored at −20 °C until further use.

### 2.4. Quantitive PCR Estimation of Kombucha SCOBY Microbial Population Size

To estimate the size of the bacterial and fungal populations within sectioned SCOBY samples, quantitative real-time PCR (qPCR) was performed. Bacterial DNA was quantified by amplifying the 16S rRNA gene, and fungal DNA by amplification of the internal-transcribed spacer (ITS) region of the 26S rRNA gene.

Bacterial qPCR was performed using 926f (5′-AAACTCAAKGAATTGACGG-3′) and 1062r (5′-CTCACRRCACGAGCTGAC-3′) primers [29], with amplification conditions as follows: 5 min @ 95 °C, then 40 cycles (15 s at 95 °C, 15 s at 61.5 °C, 20 s at 72 °C) and a final extension for 5 min at 72 °C.

Fungal qPCR was performed using yeast-F (5′-GAGTCGAGTTGTTTGGGAATGC-3′) and yeast-R (5′-TCTCTTTCCAAAGTTCTTTTCATCTTT-3′) primers [30], with amplification conditions as follows: 10 min at 95 °C, then 40 cycles (15 s at 95 °C, 60 s at 60 °C, 30 s at 72 °C) and a final extension for 5 min at 72 °C.

Each reaction contained 12.5 μL KAPA SYBR^®®^ FAST Master Mix (Roche, St. Louis, MO, USA), 0.2 μL of each primer (10 mM), and 4 μL of template DNA. PCR amplifications and product quantifications were performed using the ABI PRISM^®®^ 7500 FAST Sequence Detection System (Thermo Scientific, Waltham, MA, USA).

Liquid cultures of *B. bruxellensis* and *G. oxydans* were used to create a dilution series that covered the range of CFU/mL 10^8^–10^3^ and 10^9^–10^3,^ respectively. CFU ranges were chosen to span the range of Ct values observed in qPCR analysis of preliminary samples (data not shown). DNA was extracted from enumerated cultures and used to establish quantitative standard curves for bacterial and fungal DNA. Samples with Ct values found outside of these standard curve ranges were excluded from the analysis.

### 2.5. Metabarcoding Analyses of Kombucha SCOBY Bacterial and Fungal Communities

**Figure 1:**
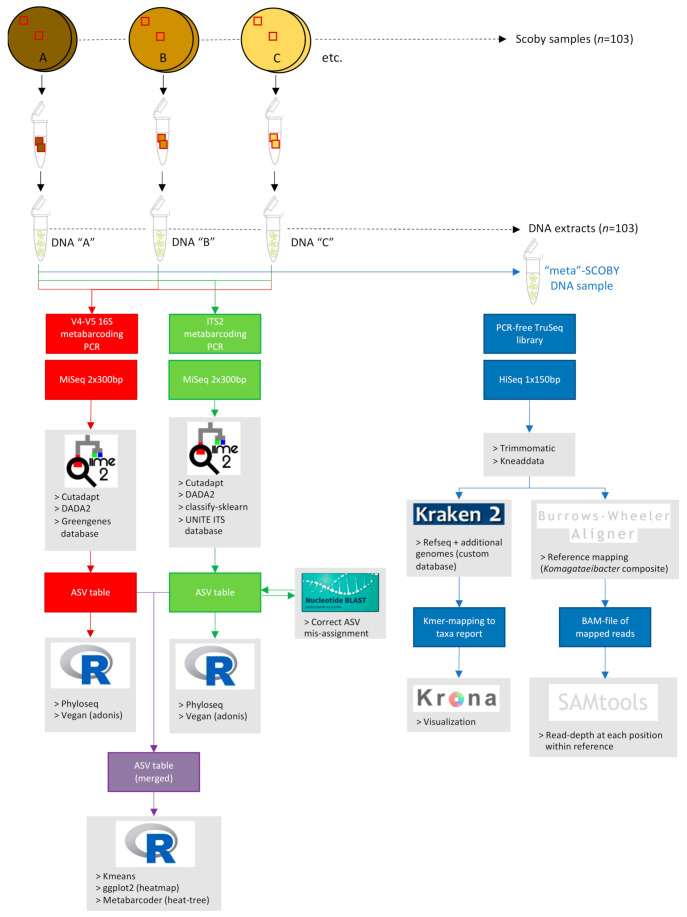
Schematic overview of metabarcoding and shotgun metagenomic sequencing library preparation and data analysis pipelines, as performed for Taxonomic Diversity Study. Metabarcoding of Spatial Analysis Study samples was performed using the same pipeline.

A schematic overview of metabarcoding library preparation and analysis of sequencing data is shown in Figure 1.

#### 2.5.1. Metabarcoding Library Preparation and Sequencing

Libraries for metabarcoding analyses were prepared as described by Comeau et al. [31], with minor modifications. For analysis of fungal communities, the fungal ITS2 region was amplified using BITS-F and B58S3-R primers [32]. For analysis of bacterial communities, the bacterial 16S V4–V5 domain was amplified using F515 and R926 primers. Forward and reverse fusion primers were designed using the scheme recommended by Comeau et al. (2017) for Illumina Nextera XT v2 indices and Nextera adapters (Table S2).

Each DNA extract was amplified twice (reactions using 1× and 1/10× DNA concentration) using 5 µL of Platinum Hot Start 2× master mix (Thermo Scientific, Waltham, MA, USA), 1 µM of each primer, 2 µL of template DNA, and molecular biology grade water (Biotium, Hayward, NJ, USA) in a final volume of 25 µL. Successful amplification of DNA templates was verified by visualizing products on 2% agarose gels using 6× GelRed Nucleic Acid Stain (Biotium, Hayward, NJ, USA). Duplicate products were combined, and then purified and normalized using SequalPrep Normalization Plates (Thermo Scientific, Waltham, MA, USA) according to manufacturer’s instructions. Pooled libraries were quantified using the Qubit dsDNA HS assay (Thermo Scientific, Waltham, MA, USA), and median fragment size determined on the Bioanalyzer 2100 high sensitivity DNA assay (Agilent, Santa Clara, CA, US). The pooled fungal ITS2 and bacterial 16S libraries were sequenced separately using MiSeq 2 × 300 bp v3 chemistry by the Oregon State University Centre for Genome Research and Biocomputing (CGRB, Corvallis, OR, USA), with a 5% PhiX spike-in, according to standard Illumina protocols.

#### 2.5.2. Metabarcoding Sequence Processing and Analyses

Sequence processing was performed by following the Microbiome Helper workflow [31] and the QIIME2 v2020.12 tutorial [33].

Demultiplexed forward and reverse FASTQ files were processed with QIIME2 plugin Cutadapt version 2020.2.0 [34] and subsequently passed through DADA2 [35] to denoise, filter chimeras, trim low-quality bases, and form an amplicon sequence variant (ASV) table. Taxonomy was assigned at the species and genus level to each ASV feature by alignment to the 16S Greengenes version 13_8 (2013) [36] and fungal ITS UNITE version 7.0 (2018) databases [37] using the taxa-barplot QIIME2 plugin [38]. The UNITE database for fungal identification was trained using a Naïve Bayes classifier and the QIIME2 classify-sklearn plugin (Pedregosa, 2011) [39].

Unassigned ITS features with a relative abundance of >0.5% across all SCOBY samples were further evaluated by performing blastn queries [40] against the NCBI nr/nt database. Where evident that adapter contamination had resulted in failed taxa assignment, top-hit information was utilized to generate a modified ASV table where read counts for these features were combined with read counts from correctly assigned taxa. ASV assignments for the ZymoBiomics DNA and extraction community standards were compared (Table S3).

To reduce rare ASVs in the dataset, ASVs with relative abundance < 0.01% of the all-sample average were removed unless at least three samples had a relative abundance of >0.01% or if at least one sample had a relative abundance of >0.05%.

### 2.6. Shotgun Metagenomic Sequencing Analysis of Composite ‘Meta-’SCOBY DNA Sample

A schematic overview of shotgun sequencing library preparation and analysis of sequencing data is shown in Figure 1.

#### 2.6.1. Meta-SCOBY Library Preparation and Sequencing

A composite ‘meta’-SCOBY was made by pooling equimolar amounts of DNA from each of the 103 individual Kombucha SCOBY DNA extracts. This synthetic representative sample was prepared for sequencing by CGRB using the TruSeq Nano PCR-free kit (Illumina, San Diego, CA, USA), with DNA shearing performed using the S2 focused ultrasonicator (Covaris, Woburn, MA, USA) as suggested in the Illumina protocol. Library quality was assessed using the Agilent 2200 TapeStation (Agilent Technologies, Santa Clara, CA, USA) and a Qubit 2.0 fluorimeter (Invitrogen, Carlsbad, CA, USA). The meta-SCOBY library was sequenced using one Illumina HiSeq 3000 1 × 150 bp lane (Illumina, San Diego, CA, USA) by the CGRB.

#### 2.6.2. Sequence Pre-Processing

The raw meta-SCOBY FASTQ file was pre-processed using Kneaddata v. 0.5.1 (http://huttenhower.sph.harvard.edu/kneaddata (accessed on 24 July 2019)), which retained 262,736,056 (94.28%) of reads following quality-trimming with Trimmomatic v0.36 [41] (parameters: SLIDINGWINDOW:4:20, MINLEN:50) and 262,635,245 (99.96%) of trimmed reads after mapping using bowtie2 [42] against GRCh38_PhiX to remove contaminant sequences.

#### 2.6.3. Kmer Analysis of Meta-SCOBY Community Composition

Community composition of the meta-SCOBY was assessed by kmer hashing using Kraken2 [43]. Initial analysis suggested that a large number of kmers were not being assigned to taxa that were expected to be present based upon metabarcoding analysis. An updated kraken2 database was constructed, incorporating the accessions provided in Supplementary Table S4. Kraken2 output was visualized using Krona [44].

#### 2.6.4. Mapping of Meta-SCOBY Reads against a Komagataeibacter Composite Reference Genome

In order to resolve ambiguous kmer mapping against the *Komagataeibacter* genus, a composite reference genome was constructed using reference genomes for *K. cocois, K. europeus, K. hansenii, K. medellinensis, K. nataicola, K. pomaceti, K. rhaeticus, K. saccharivorans* and *K. xylinus* (Table S4). The kneaddata pre-processed fastq was mapped against this reference using bwa-mem [45], and average read depth per reference genome calculated using samtools depth [46].

### 2.7. Statistical Analyses

Metabarcoding data visualization was performed in R v4.0.2 following the Phyloseq tutorial (https://vaulot.github.io/tutorials/Phyloseq_tutorial.html (accessed on 9 April 2019)) [47]. Species richness (Observed, Chao1), alpha diversity (Shannon, Simpson, Inv), and beta diversity metrics were grouped by facets of “Layer”, “Origin”, and “Cluster” variables. Beta diversity was calculated by Bray–Curtis distance and visualized using ggplot2 [48]. The number of clusters, based upon combined fungi and bacteria (genus-level) ASV relative abundances, was determined by kmeans. Generalized UniFrac and weighted UniFrac scores were calculated using the phyloseq distance function and were subsequently used to determine the dissimilarity between samples according to their bacterial and fungal communities [49]. Phylogenic heat trees of prevalent taxa (relative abundance >0.1% in at least 3 samples or >0.5% in one sample) for each cluster were drawn using Metacoder [50]. Permutational multivariate analyses of variance (PERMANOVA) were performed using adonis in vegan [51].

Comparisons of alpha diversity, relative abundances of individual taxa and qPCR estimates of fungal and bacterial abundances, were performed in R using standard one- or two-way ANOVA, along with Student’s t-tests. Comparison of taxa relative abundance between metabarcoding and shotgun sequencing kmer datasets was performed by construction of linear models in R.

All R code to reproduce statistical analyses are available as an R markdown file at https://github.com/curtinlab/SCOBY diversity (accessed on 7 April 2021).

### 2.8. Sequence Data Availability

Fastq files available at NCBI BioProject: PRJNA719546.

## 3. Results

### 3.1. Spatial Distribution of Fungi and Bacteria within a Single Kombucha SCOBY

To better understand the distribution of microbial communities within the solid-phase SCOBY (and guide SCOBY sampling strategies), a representative SCOBY previously used to commercially brew Kombucha was divided into two layers and dissected radially. qPCR analysis revealed significant differences in total abundance of fungi and bacteria between upper and lower layers, along with significant differences in the composition of these communities.

**Figure 2:**
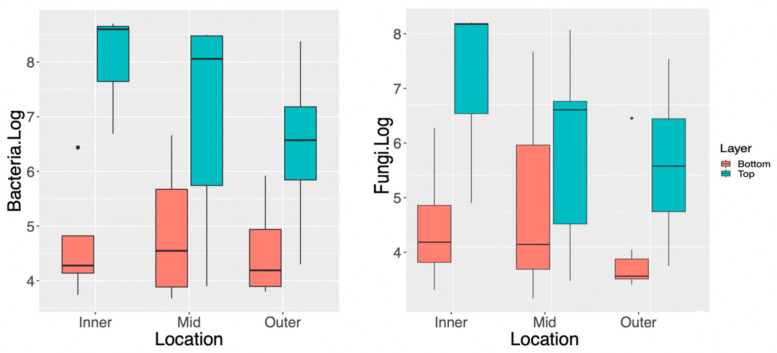
Spatial distribution of bacteria and fungi within sectioned Kombucha SCOBY. Quantitative real-time PCR ct-values converted to log-CFU/g against standard curves of *Brettanomyces* (fungi) and *Gluconobacter* (bacteria).

As shown in Figure 2, the top layer of this SCOBY was more enriched than the bottom layer for both fungi and bacteria, regardless of radial sampling position, with approximately 3-log more (3.55 × 10^7^ CFU/g versus 3.02 × 10^4^ CFU/g, respectively) bacteria and 2-log (3.55 × 10^7^ CFU/g versus 3.02 × 10^4^ CFU/g, respectively) more fungi. Two-way ANOVA reinforced this observation; radial position was not significantly different (bacteria *p* = 0.28, fungi *p* = 0.31), whereas layer was significantly different (bacteria *p* = 4.26 × 10^−5^, fungi *p* = 9.08 × 10^−3^, Table S5).

**Figure 3:**
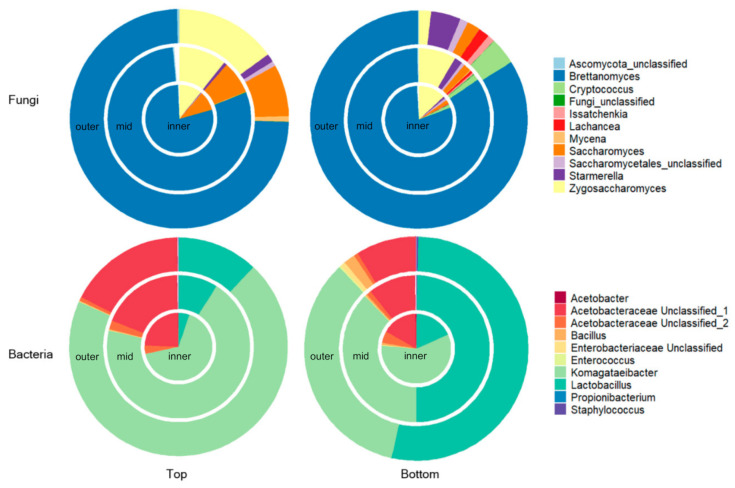
Spatial variation in the composition of fungal and bacterial communities within sectioned Kombucha SCOBY, derived by metabarcoding analysis. Percentages of normalized sequencing reads for each taxa are presented within concentric rings according to their spatial position (inner, mid, outer), with separate plots for top and bottom SCOBY layers.

Metabarcoding of the sectioned SCOBY revealed a microbial community dominated by fungal genera *Brettanomyces* and *Zygosaccharomyces,* and bacterial genera *Komagataeibacter* and *Lactobacillus* (Figure 3). Similar to the qPCR results, PERMANOVA analysis of beta diversity shows a significant difference in composition of bacterial and fungal communities according to layer (bacteria *p* < 0.001; fungi *p* = 0.025) but not radial position (bacteria *p* = 0.255; fungi *p* = 0.790) (Table S6). Interestingly, the fungal taxa that varied significantly (*p* < 0.05) in relative abundance according to layer were relatively minor taxa of the SCOBY (*Saccharomyces, Issatchenkia, Lachancea, Cryptococcus*) whereas for bacteria significant differences were evident for the major genera (*Komagataeibacter* and *Lactobacillus*) (*p* < 0.001, Table S6). Notably, reads assigned to *Lactobacillus* comprised 44.2 ± 29.1% of the bacterial reads for the bottom layer of this SCOBY, compared with 9.7 ± 7.9% of bacterial reads in the top layer. Within the bottom layer, mean relative abundance of *Lactobacillus* and *Komagataeibacter* appeared to vary according to location (inner, mid, outer). However, PERMANOVA analysis on overall beta diversity (*p* = 0.234) and all pairwise *t*-test comparisons of relative abundance by location for *Lactobacillus* and *Komagataeibacter* were non-significant (*p* > 0.05).

### 3.2. Fungal and Bacterial Communities Associated with 103 Commercial Kombucha SCOBY

In order to generate global species-level information on the major taxa observed in commercially used Kombucha SCOBY cultures, a composite DNA ‘meta’-SCOBY was assessed with a shotgun sequencing approach. The same 103 samples included within the ‘meta’-SCOBY were also assayed individually using 16S and ITS metabarcoding, to facilitate analysis of taxa prevalence and clustering of samples according to their overall metabarcoding profiles (see analysis schematic in Figure 1).

**Figure 4:**
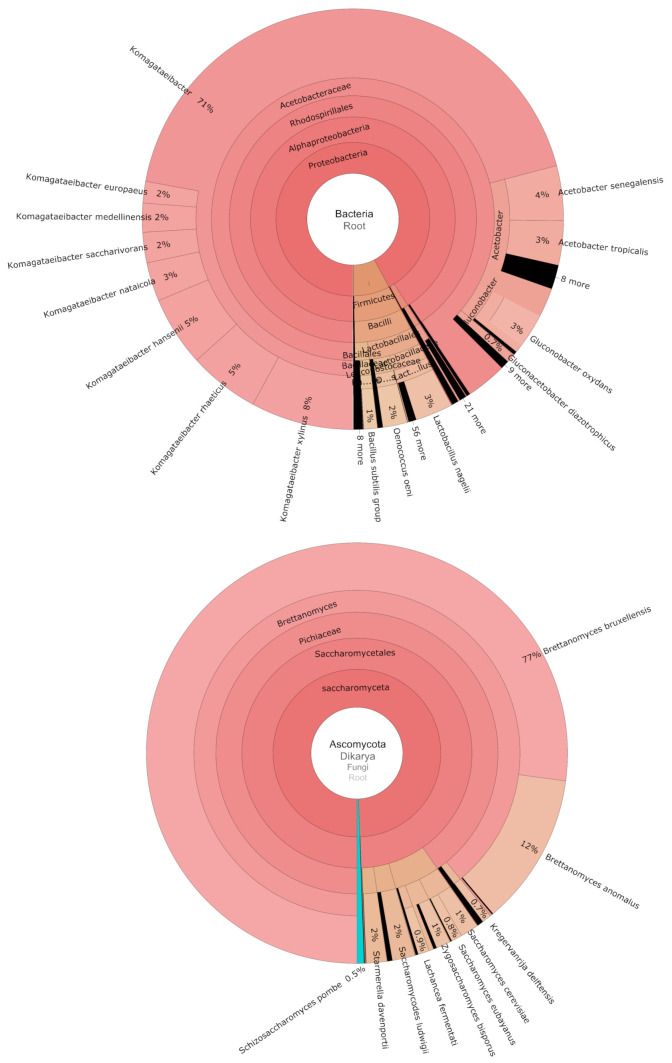
Shotgun sequencing of composite ‘meta’-SCOBY. Results of Kraken2 kmer analysis presented as Krona charts for bacteria and Ascomycota, which made up 99.5% of fungal reads.

Of the 176,454,543 km derived from shotgun sequencing of the meta-SCOBY (comprising equimolar DNA from all 103 SCOBY samples), 61.5% mapped to bacterial taxa, 27.3% to fungi, and 11.1% remained unmapped. Prior to incorporation of additional reference genomes to a custom Kraken2 database, a greater portion (36.9%) of kmers were unmapped, mostly due to fungi that were missing or poorly represented. As observed for the dissected SCOBY (described in Section 3.1), *Brettanomyces* and *Komagataeibacter* were by far the dominant genera (Figure 4, Table S7). Amongst the ten species assigned > 0.5% of fungal kmers, *Brettanomyces bruxellensis* and *B. anomalus* were represented by approximately 76.9% and 11.7%, respectively. Surprisingly, the relative abundance of *Zygosaccharomyces bisporus* was much lower (1.1%). Indeed, more kmers were mapped to both *Starmerella davenpoortii* and *Saccharomycodes ludwidgii* (both 1.7%).

For bacteria, there were sixteen species assigned > 0.5% of kmers; 70.8% corresponding to *Komagataeibacter*, with the next most abundant genera being *Acetobacter* (11.7%), *Gluconobacter* (3.5%), and *Lactobacillus* (3.5%). Interestingly, the majority of kmers mapped to *Lactobacillus* were assigned to *L. nagelii* (3% of total bacterial kmers). Whilst the vast majority of kmers were resolved to species level, this was not the case for some bacterial genera, particularly *Komagataeibacter*. To determine which species from this dominant genus were most abundant in the ‘meta’-SCOBY, full-length reads were mapped against a composite reference comprising representative genomes of each *Komagataeibacter* species. Approximately 13.5 Gbp of shotgun sequencing data mapped against this reference (Table S8), which represents ~34% of full length reads. Considering only a single assembly was used for each species, this corresponds reasonably well with kmer analysis, where 43.6% of all kmers were assigned to *Komagataeibacter*. Reference mapping showed that while all nine species included in the composite reference were represented in the meta-SCOBY sample, they were not equally abundant (Table S8). Based upon total reads mapped, 64% corresponded to *K. rhaeticus*, whereas the next most represented species, *K. xylinus*, only received 8% of mapped reads.

Metabarcoding analysis of the 103 SCOBY samples revealed average relative abundances of key taxa that were correlated (*p* = 0.016) with those found by kmer analysis of the meta-SCOBY shotgun sequencing reads (Table 1 and Table S9). There were, however, some differences between the datasets. For example, *Lactobacillales* was approximately twice as abundant (12.9% compared to 5.9%) according to 16S metabarcoding, while *Acetobacter* was around 10-fold less abundant (1.2% compared to 11.7%). *Tanticharoenia* was substantially underrepresented (by ~100 fold) in the kmer dataset. It is worth noting that the single available reference genome for this genus (added to our custom Kraken2 database) may not be sufficiently representative of the taxa detected by metabarcoding of the 16S region.

Metabarcoding data was further analyzed to examine prevalence of the major genera and minor contributing taxa. *Komagataeibacter* and *Brettanomyces* were detected in 97% and 99% of the samples, respectively. Less abundant fungal taxa *Zygosaccharomyces, Starmerella*, and *Lachancea* were detectable at greater than 0.1% abundance in 39–63% of samples. Likewise, unidentified *Lactobacilliacae* and Lactobacillales contributed to only 8.0% of total reads across all samples, yet both were detected in approximately one-third of samples.

### 3.3. Defining Kombucha SCOBY Archetypes Based upon Microbial Community Composition

Bacterial and fungal ASV tables were merged into a single dataset to facilitate identification of SCOBY archetypes based upon overall microbial composition. First, alpha diversity was evaluated. The average observed number of ASVs was determined to be 4.68 ± 1.67 taxa (Table S10). This level of taxa richness is relatively low when considering that 12 of the 19 ASVs (Table 1) were prevalent in >10% of samples. Alpha diversity (Simpson Index, SI) was observed as consistent across samples regardless of collection time frame (Batch 1 or 2) or culture origin (North America or Other), suggesting that these factors did not have an impact on species richness (Table S10).

**Figure 5:**
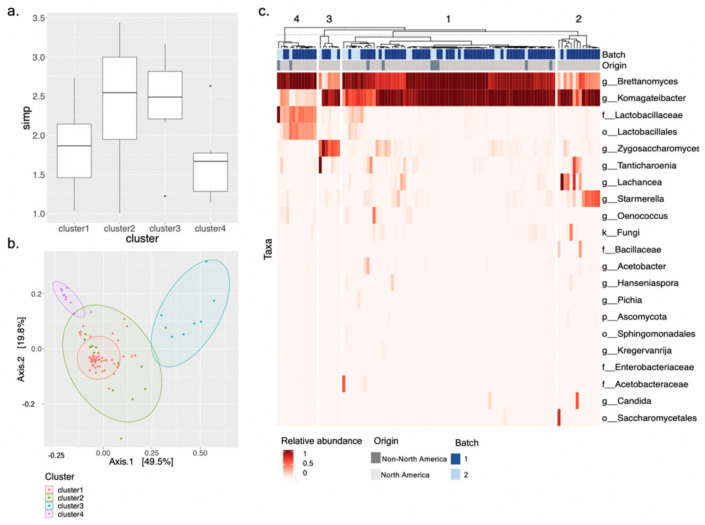
Microbial community composition of 103 commercial Kombucha SCOBY determined by metabarcoding analysis and divided into four k-clusters. (**a**) Comparison of Simpson Diversity Index. (**b**) Ordination plot of weighed Unifrac distance comparing beta diversity. (**c**) Two-way hierarchically clustered heatmap showing the relative abundance of bacterial and fungal microbial genera within individual SCOBY, grouped by similarity and divided into four k-clusters.

Unsupervised K-means clustering was then used to cluster SCOBY samples by their microbial composition. Based upon “elbow” and “cluster” methods, the optimal numbers of k-clusters were two and four (Figures S2 and S3). A two-way hierarchically clustered heat map shows that with k = 2, SCOBY samples were clustered almost entirely based upon relative abundance of *Brettanomyces* (Figure S4). With k = 4, delineation of SCOBY archetypes according to relative abundance of other fungal and bacterial taxa can be observed (Figure 5c). PERMANOVA was used to confirm that sample beta diversity differed significantly according to k-cluster designation (F = 36.94, *p* = 0.001) (Figure 5b, Table S11). The same analysis showed that, as was the case with alpha diversity, beta diversity was not significantly different between samples grouped by collection time frame (*p* = 0.167) or culture origin (*p* = 0.392). Indeed, it was notable that the SCOBY harvested outside of North America were distributed proportionally across the four k-clusters (Table S11).

Correction for possible oversampling of SCOBY from larger producers (“thinned” dataset—repeat submissions from producers removed if assigned to same k-cluster) had no effect on cluster composition with regard to relative abundance of key taxa, prevalence of key taxa, and overall beta diversity (Tables S11–S13).

Looking more closely at diversity within the k-cluster designations, we observed alpha diversity (SI) to vary significantly by clusters but not by the number of taxa observed. Cluster III had the smallest number of observed taxa, 4.00 ± 0.58, followed, respectively, by Clusters I, II, and IV (Table S10). The number of observed taxa does not account for species richness or evenness of abundance, so the Simpson’s index was used to more accurately compare biodiversity among the clusters. Cluster I has significantly less biodiversity when compared to the other clusters (Figure 5a, Table S10). From the heatmap, we observe Cluster I to be dominated by *Brettanomyces* and *Komagataeibacter* (Figure 5c). The remaining clusters exhibit various combinations bacterial or fungal taxa in place of the diminished abundance of *Brettanomyces* or *Komagataeibacter*. Cluster II is comprised of *Komagataeibacter* with low to moderate abundance of *Brettanomyces* and elevated abundance of either *Starmerella* or *Lachancea*. Cluster III is also *Komagataeibacter* dominant, with low to moderate abundance of *Brettanomyces* and high abundance of *Zygosaccharomyces*. Lastly, Cluster IV is *Brettanomyces*-dominant with a low abundance of *Komagateibacter* and a high abundance of *Lactobacillales*.

**Figure 6:**
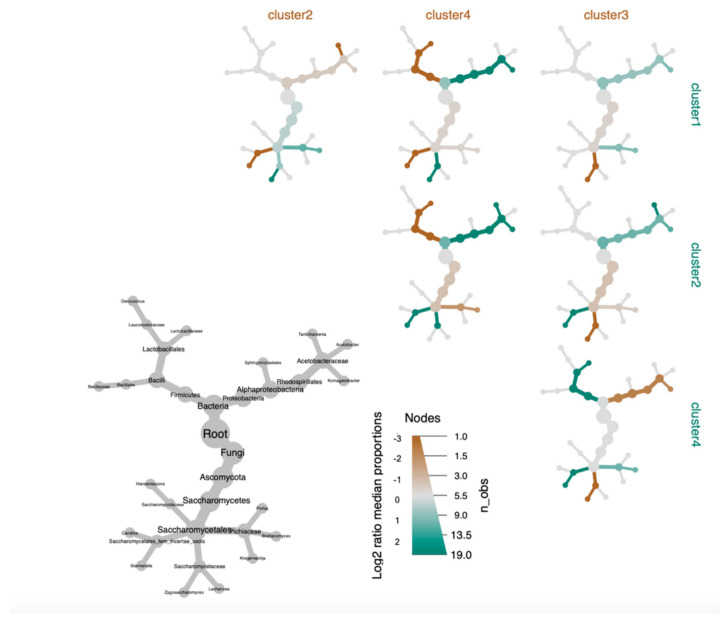
Differential phylogenic heat tree of ASV relative abundance for each cluster. Tree includes ASVs that differ significantly in median read proportion (log2 ratio of median proportions) between k-cluster groupings of 103 commercial Kombucha SCOBY. Differences depicted as greater abundance in row clusters (green) versus column cluster (brown). Significance was determined using Wilcox rank-sum tests with a false discovery rate correction for multiple comparisons.

In order to more clearly visualize nuanced differences between clusters, a phylogenic heat tree matrix was constructed for taxa that significantly differed in relative abundance between clusters (*p* < 0.05) (Figure 6). As was observed in the heatmap, mean abundance of *Komagataeibacter* in Cluster I (*n* = 69, 66.9%) was higher than for Clusters III and IV. However, *Komagataeibacter* was lower in Cluster I than in Cluster II (*n* = 14, 13.6%). *Starmerella* appears to cooccur in Cluster II with a higher abundance of *Komagataeibacter.* Interestingly, when looking at the heatmap and heat tree together, we are able to observe that within cluster II, *Tanticharoenia* is the dominant bacterium when *Starmerella* is not as abundant. We potentially observe an antagonistic across-Kingdom dynamic between *Starmerella* and a specific genus of AAB.

*Lactobacillaceae*, prevalent in 100% of Cluster IV samples (*n* = 13, 12.6%) and significantly more abundant (*p* < 0.001) relative to other clusters, was only found in approximately 29–30% of the samples in the remaining clusters (Tables S13 and S14). From the heat tree, we observe that *Lactobacillaceae* and *Komagateibacter* negatively co-occur in Cluster IV. Consistent with this observation, *Komagateibacter* is significantly (*p* < 0.001) less abundant in Cluster IV with a decreased abundance of *Lactobacillaceae*.

When looking at differences in the fungal taxa between the clusters in the heat tree, we note a higher abundance of *Brettanomyces* in clusters when there is a lower abundance of either *Zygosaccharomyces* or, collectively, *Lachancea* and *Starmerella*. Cluster III (*n* = 7, 6.8%) has a significantly higher (*p* < 0.001) abundance and substantially greater prevalence of *Zygosaccharomyces* when compared to the remaining clusters (Tables S13 and S14). When comparing Cluster III to Cluster I, we see substantial decrease in *Brettanomyces*. Meanwhile, Cluster II has lesser abundance of *Brettanomyces* and comparatively higher abundances of either *Lachancea* (*p* < 0.01) or *Starmerella* (*p* < 0.001) than Cluster I. Collectively, structural differences among the clusters revealed a broad inverse relationship between the abundance of *Brettanomyces* and both *Starmerella* and *Lachancea*. When *Starmerella* and *Lachancea* are not present, *Zygosaccharomyces* seems to compensate for a decreased abundance of *Brettanomyces*.

## 4. Discussion

Fermented foods and beverages, when subject to minimal human intervention, are dynamic ecological systems characterized by initial assembly of complex microbial communities that subsequently collapse into predictable structures through biotic and abiotic interactions. These dynamic changes are well understood for some fermentations, such as Lambic beer [52,53], wine [54,55,56] and cheese [57,58]. Efforts to understand the microbial ecology of Kombucha have revealed a greater degree of apparent interchangeability in dominant yeast and bacterial species [22]. Better understanding of microbial community assemblies found in Kombucha SCOBY would facilitate further research into drivers of assembly patterns, and their relevance to Kombucha style.

A significant challenge in profiling the microbial community composition of SCOBY is adequately sampling this cellulosic solid-phase material. Indeed, very few studies have described the sampling scheme used to characterize the Kombucha SCOBY. Regardless of the downstream analytical approach, there is generally a failure describe how the SCOBY was handled prior to culture plating or DNA extraction [14,16,17,59]. Of the four studies that described sample processing, there was brief mention of excising a small section from a larger SCOBY, followed by homogenization or enzymatic digestion [1,5,8,60]. Furthermore, there was no detailed mention of a methodical scheme to ensure representative sampling, but rather a presumption of spatial homogeneity. Our results show that if sampling does not include upper and lower layers of the SCOBY, significant differences in abundance and community composition may be incorrectly inferred.

The idea that biochemical properties of the environment contribute to spatial variability within a microbial community is not new [61]. In Lambic beer fermentations, Roos et al. [62] found higher AAB counts correlated with higher metabolic activity (increased production of acetic acid, acetoin, and ethyl acetate) at the air/liquid interface. Given that SCOBY typically float to the surface of fermenting Kombucha, they represent an interphase environment, where oxygen availability, temperature and nutrient availability vary on either side, parameters likely to influence solid-phase formation and microbial community assembly [63,64,65]. The major taxa observed in the dissected SCOBY, *Brettanomyces* and *Komagataiebacter*, both have a strong affinity for oxygen [66,67], thus it makes sense that the upper layer harbored significantly larger fungal and bacterial communities. Likewise, greater abundance of *Lactobacillus* in the lower SCOBY layer is consistent with known properties of *Lactobacillus nagelli* (growth preference in high nutrient, low oxygen environments) [68], which we observed as the most abundant Kombucha SCOBY LAB in shotgun sequencing analysis.

Estimates of relative abundance for some important Kombucha taxa, such as the bacterial order Lactobacillales and fungal genera *Zygosaccharomyces*, differed between the shotgun sequencing kmer analysis of a synthetic ‘meta’-SCOBY, and averaged results of metabarcoding for each individual SCOBY. This may be explained by underrepresentation of relevant species in the RefSeq database Kraken2 draws upon [69] or inherent biases in amplification of marker gene sequences targeted in metabarcoding analysis [70]. While we did add several whole-genome assemblies to a custom database, which did improve the proportion of mapped kmers, it is likely the fidelity of mapping will improve as more Kombucha-relevant organisms are sequenced. Regardless, the ‘meta’-SCOBY kmer analysis generally agreed with metabarcoding results for major genera, elucidating the main fungal and bacterial species across 103 Kombucha SCOBY. An additional limitation of kmer analysis was evident for *Komagataeibacter*, where most 35 bp kmers could not be assigned at species level. We augmented this analysis with composite-reference mapping of full-length sequencing reads to determine that *Komagataeibacter rhaeticus* was by far the most represented species.

Despite potential artifacts regarding sampling in prior studies, three of the four Kombucha SCOBY archetypes we defined here are consistent with those previously described. Comparing only to studies that used molecular-based approaches, Gaggia et al. [8] and Reva et al. [4] both described profiles consistent with the Cluster I archetype. Profiles that resemble Cluster III, with an increased abundance of *Zygosaccharomyces* [14,71] and SCOBY with greater abundance of *Lactobacillales* (Cluster IV) [16] have also been reported. Prior to this study, *Starmerella*, one of the major fungi replacing *Brettanomyces* in Cluster II, had not been described in molecular ecology studies of the solid-phase Kombucha SCOBY. *Starmerella* is a relatively new genus into which several *Candida* species have been reclassified [72], thus earlier Kombucha studies reporting *Candida* at genus level may have observed species currently classified as *Starmerella*. *Starmerella davenportii*, detected in the ‘meta’-SCOBY, was recently isolated from Kombucha and shown to generate volatile aroma compounds and organic acids during a black tea fermentation [73].

It is interesting to note that while *Brettanomyces* is observed in our data to be the most abundant yeast genus in three of the four SCOBY archetypes, other studies of Kombucha ecology typically report it as a non-dominant feature amongst genera such as *Saccharomyces*, *Zygosaccharomyces*, and *Pichia* [1,13,74,75]. To date, *Zygosaccharomyces* has been more frequently described as the dominant Kombucha yeast genus [1,13,14,71,75,76]. Given that prior works have sampled from relatively few individual SCOBY this may simply reflect under-sampling, but because most of our samples originated from Kombucha producers in North America an alternative explanation is that SCOBY geographic origin influences microbial community structure. Terroir, the concept of regional identity most strongly associated with wine production, has increasingly incorporated biogeographical patterns of microbial diversity [77]. In other fermentation systems, such patterns are less apparent. Wolfe et al. [25] demonstrated that cheese rinds from geographically diverse areas (Europe and North America) have similar microbial communities, finding that environmental factors were more important as drivers of community divergence. Likewise, assembly of similar microbial communities have been noted for sour-beer fermentations in Belgium and the United States [53,78]. While we analyzed only a small number (11) of SCOBY from outside of North America, these samples were not outliers and in fact displayed similar microbial community structures to the North American SCOBY. This observation reinforces that while reproducible Kombucha SCOBY communities may assemble across diverse geographical regions, there are more divergent SCOBY community structures or archetypes than described for related fermentation systems.

A potential artifact that should be recognized is the possibility that across different studies SCOBY were sampled at different points during Kombucha fermentation. Within our study, while SCOBY were sampled at the end of fermentation it is worth noting that different Kombucha producers define their end-points differently according to their desired sweet-acid balance. As a dynamic fermentation system, it is reasonable to expect relative abundance of taxa to change over time. For example, Chakravorty et al. [17] observed a dominant *Candida stellimalicola* population at the start of fermentation with an increasing abundance of *Lachancea fermentati* as fermentation progressed. While this shift in abundance occurred in both the solid and liquid phases, the magnitude was substantially greater in the liquid. Relative abundance of *L. fermentati* increasing from 15.5% on day 3 to 51% on day 7, whereas in the SCOBY *L. fermentati* increased from 2.3% to 2.5% from day 3 to day 7. Thus, while we cannot exclude that the SCOBY archetypes we observed could be a function of how individual producers define end of fermentation, the SCOBY itself can be considered buffered and relatively stable with regard to microbial composition. Similar observations were made by Teoh et al. (2004) [1], where *B. bruxellensis* was observed to stabilize in the solid phase from 10^8^ cfu/g on day 4 (following biofilm formation) to 10^8^–10^9^ cfu/g on day 10. Meanwhile, in the liquid phase, *B. bruxellensis* population increased from approximately 10^4^–10^5^ cfu/g at day 4 to 10^7^ cfu/g at day 7. These observations are consistent with the widely accepted tenant that biofilm formation is a survival mechanism that provides microorganisms with greater environmental stability [79,80]. Given this background, the stability of the microbial composition of SCOBY is promoted by the biofilm formation in the pellicle layer. Furthermore, because the SCOBY is serially transferred from completed to new batches of Kombucha, it is likely the microbial community it harbors would stabilize according to the physicochemical parameters of these conditions. Parallels to this can be observed in the gradual stabilization of sourdough cultures [81]. It is also worth noting that Kombucha producers operate across a range of scales, utilizing diverse fermentation equipment. The most obvious way this may affect SCOBY community structure is the availability of dissolved oxygen, given the reliance upon this nutrient for AAB [82] and the known stimulatory effect it has on *Brettanomyces* fermentation rate [83]. Cvetkovic et al. [84] demonstrated that altering the surface-to-volume ratio in open fermenters had a significant effect upon rate of Kombucha fermentation. While community structure was not described in that study, it seems reasonable to expect that the conditions would favor certain SCOBY archetypes over others.

As an acidic beverage, organic acids are pivotal to the organoleptic properties of Kombucha. Different organic acids possess unique sensory attributes such as flavor and thresholds of detection; for example, lactic acid is perceived as ‘tart’ and ‘acrid’ and is detectable at 400 mg/L, while acetic acid perceived as ‘vinegar’ and ‘sour’ is detectable at 180 mg/L [85,86,87,88]. Cluster II SCOBY, with high proportions of lactic acid bacteria, might be reasonably expected to produce more lactic acid during Kombucha fermentation, altering organoleptic properties of finished Kombucha. Likewise, SCOBY composition may affect production of acetic acid. In the presence of oxygen, *Brettanomyces* yeasts contribute to acetic acid production [89] alongside the more obvious contribution from acetic acid bacteria [90,91]. In support of this, Tran et al. (2020) observed that Kombucha fermentations performed by co-cultures of *Hanseniaspora valbyensis* and *Komagataeibacter saccharivorans* contained less sucrose phosphorylase activity than fermentations performed by *Komagataeibacter saccharivorans* and *Brettanomyces bruxellensis*, and were characterized by decreased production of both acetic and lactic acids [22].

Beyond organic acids, the impact of biodiversity on the quality and flavors of fermented products is a well-studied phenomenon [92,93]. For example, the contribution to wine aroma and flavor by non-*Saccharomyces* yeasts, such as *Lachancea* and *Torulasporula*, occurs through the direct biosynthesis of volatile aroma compounds and a large variety of molecules, including volatile fatty acids, higher alcohols, esters, and sulfur compounds [94]. Meanwhile, volatile phenolic compounds are responsible for the most recognized aromatic impacts associated with *Brettanomyces* species. ‘Brett flavors’ are considered spoilage in wine and have been described as ‘barnyard’, ‘clove’, ‘horsy’, ‘leathery’, and ‘medicinal’ [95]. Furthermore, in wine it has been shown that *Brettanomyces* and heterofermentative lactic acid bacteria cause a form of spoilage known as ‘mousy’ taint, through biosynthesis of nitrogen heterocyclic pyridines from lysine and ethanol [95]. In the context of beer, certain styles such as Lambic, Gueze and American Coolship Ale, the impact of *Brettanomyces* upon flavor is viewed more positively [96]. It is unclear to what extent *Brettanomyces*-related flavors are observed in Kombucha, or whether consumers find them desirable in this context, but the distinction between SCOBY archetypes according to presence of *Brettanomyces* makes it reasonable to speculate a potential role for these yeasts in organoleptic differences between products.

## 5. Conclusions

The results of this study show that the microbial community of a Kombucha SCOBY used in commercial Kombucha production was differentiated spatially. Based upon overall abundance of fungal and bacterial taxa, as well as compositional differences, a sampling strategy that captures upper and lower layers of SCOBY is essential to ensure observed microbial communities are representative. Subsequent analyses of 103 Kombucha production SCOBY cultures, sampled in this manner, provided the first comprehensive picture of SCOBY microbial community assembly. While there was no evidence of geographic influence upon fungal or bacterial community composition, the datasets were used to delineate four SCOBY archetypes. Based upon prevalence and relative abundance, we find that the major taxa amongst North American SCOBY belong to the genera *Brettanomyces* and *Komagataeibacter*. SCOBY archetypes comprised of other taxa noted previously in Kombucha literature were also evident, but were far less common.

Further research is necessary to relate the microbial community composition of Kombucha SCOBY to acidity, flavor and aroma of finished products. Such work can now draw upon knowledge of SCOBY archetypes defined here, as the basis to design industry-relevant co-culture experiments that test the impact of major taxa. Comparative shotgun metagenomics of multiple SCOBY, inclusive of metabolic pathway reconstruction, would likely contribute to hypothesis formation in this context. Furthermore, given the relatively small number of dominant taxa within each SCOBY community, Kombucha fermentation represents a tractable system that can be manipulated to study the rules that govern community assembly in an interphase environment.

## Figures and Tables

**Table 1:** Average relative abundance and prevalence of fungal and bacterial ASVs across 103 Kombucha SCOBY samples as determined by metabarcoding analysis, compared with results of shotgun-sequencing of the synthetic ‘meta’-SCOBY sample.

Taxa (ASV)	Metabarcoding		Shotgun Sequencing
	Relative Abundance	Prevalence ^1^	Proportion of Kmers ^2^
*Fungi*			
g__Brettanomyces	0.813 ± 0.259	0.990	0.8884
g__Zygosaccharomyces	0.068 ± 0.170	0.625	0.0173
s__Starmerella davenportii	0.047 ± 0.124	0.481	0.016
s__Lachancea fermentati	0.038 ± 0.141	0.385	0.0086
k__Fungi	0.012 ± 0.050	0.183	NA
f__Saccharomycetales_unidentified	0.008 ± 0.072	0.019	NA
g__Candida	0.007 ± 0.062	0.019	0.0005
g__Hanseniaspora	0.004 ± 0.021	0.125	0.0038
g__Pichia	0.001 ± 0.011	0.010	0.0043
p__Ascomycota	0.001 ± 0.005	0.058	NA
g__Kregervanrija	0.001 ± 0.004	0.019	0.0076
*Bacteria*			
g__Komagataeibacter	0.809 ± 0.299	0.971	0.7088
Lactobacillales total	0.129		0.0589
f__Lactobacillaceae	0.072 ± 0.167	0.394	NA
o__Lactobacillales_unidentified	0.050 ± 0.117	0.327	NA
g__Oenococcus	0.007 ± 0.061	0.077	0.019
g__Tanticharoenia	0.039 ± 0.130	0.462	0.0003
g__Acetobacter	0.012 ± 0.055	0.173	0.117
f__Acetobacteraceae	0.006 ± 0.030	0.144	NA
f__Bacillaceae_unidentified	0.005 ± 0.040	0.029	0.0112

^1^ Proportion of SCOBY where relative abundance of taxa exceeded 0.1%. ^2^ Proportion of kmers mapped to taxa (at metabarcoding ASV-defined levels) within relevant microbial kingdom. For ASV’s unassigned at a comparable level, kmer proportions are not reported (NA).

## Data Availability

Fastq files available at NCBI BioProject: PRJNA719546.

## Supplementary Materials

The following are available online at https://www.mdpi.com/article/10.3390/microorganisms9051060/s1.
